# How should we decide how to treat the child: harm versus best interests in cases of disagreement

**DOI:** 10.1093/medlaw/fwad040

**Published:** 2023-12-05

**Authors:** David Archard, Emma Cave, Joe Brierley

**Affiliations:** School of History, Anthropology, Philosophy and Politics, Queen’s University Belfast, Belfast BT7 1NN, UK; Prof E. Cave, Durham Law School, Durham University, Durham DH1 3LE, UK; Paediatric Bioethics Centre, University College London, Great Ormond Street Institute of Child Health, NIHR Great Ormond Street Hospital Biomedical Research Centre, London WC1N 3EH, UK

**Keywords:** Best interests, Child, Children’s rights, Harm, Parental rights, Treatment

## Abstract

Where parents seek treatment for their young child that healthcare professionals cannot agree to, the High Court can determine what is in the child’s best interests. Some activists and academics seek change to impose threshold criteria that would bolster the decision-making rights of parents and reduce deference to clinicians and the courts. We defend the best interests standard against arguments that a higher threshold of ‘significant harm’ should apply. We do so from ethical, legal, and clinical perspectives. The matter is of significant moral and practical importance, especially in light of the divergence of academic opinion, the burgeoning number of cases coming before the courts and recent case law and statutory attempts to effect change. We begin by disputing ethical claims that a significant harm threshold is preferable to the best interests standard, and then we set out jurisprudential and practical arguments that demonstrate the imprudence of a significant harm threshold and defend the established yardstick of best interests.

## I. INTRODUCTION

In recent years, several writers have defended the view that in cases where there is substantive and irresolvable disagreement between parents and clinical staff as to how the former’s child should be treated, a significant harm (SH) rather than a best interests (BI) criterion should be employed for the High Court to have jurisdiction to make a declaration.^1^ A 2014 review even suggests consensus amongst bioethicists on the matter.^2^ The preference for an SH criterion is usually taken to mean that parental choices of medical treatment should prevail, provided they do not cause or risk causing the child SH. Based on the current law, however, the courts can override parental wishes if a different course of action will promote the child’s BI. As we shall see, there have been proposals to reinterpret or change the law to make SH the relevant criterion in these kinds of cases.

We will leave to one side the questions raised by disagreements *between parents*, and in what follows, we advance two central arguments. After setting out the legal context, Section I criticizes reasons given for preferring SH over BI, countering claims that the SH criterion would be preferable to the BI criterion because it would promote parental autonomy,^3^ enhance determinacy,^4^ respect reasonable pluralism,^5^ and improve consistency.^6^ In Section II, we provide a positive case for retaining the existing standard, setting out new arguments that an SH criterion would conflict with professional responsibilities established in criminal and tort law. Through an analysis of the legal test for BI, we also challenge arguments that the SH criterion, whilst requiring change to the conventional wisdom, would be consistent with clinical practice.^7^ Building on Jo Bridgemen’s assertion of the professional duty and public responsibility for child welfare,^8^ we seek to move the debate away from a rights claim on behalf of parents, demonstrating flaws in the normative arguments for an SH threshold and mounting a jurisprudential defence of the status quo.

## II. THE LEGAL CONTEXT

Arrangements for the treatment of seriously ill young children^9^ are generally reached through partnership between healthcare professionals (HCPs) and parents. Where disagreements arise, they are usually resolved through discussion. Sometimes mediation,^10^ clinical ethics committee advice^11^ or second opinions^12^ can help parties to reach a consensus.^13^ But there remains a subset of cases where disagreement cannot be resolved, and the High Court is approached to make a declaration about what course of action is in the child’s BI.^14^

The law is clear that the court will decide the matter independently, with consideration of, but not bound by, the views of HCPs,^15^ the young child (where they are able to express a view) and parents. In the 2023 case of Indi Gregory, for example, the High Court declared that certain invasive treatments sought by her parents were not in Indi’s BI. Indi was on life support and had profound and incurable disorders. The court found that the benefits of treatment were outweighed by the pain she would suffer.^16^ Several other recent cases are referred to in the course of this article, including Charlie Gard,^17^ Isaiah Haastrup^18^, Tafida Raqeeb,^19^ and Alta Fixsler^20^. Since August 2023 parents have been able to access means-tested legal aid where hospitals apply to withdraw or withhold life-sustaining treatment for their child,^21^ remedying a profound impediment to access to justice.

Whilst in practice many matters are left to parental discretion, their rights and powers are, in fact, limited by the concept of ‘parental responsibility’ set out in section 3(1) of the Children Act 1989.^22^ This has been interpreted to mean that parents do not have an absolute right to make decisions on behalf of children, that the court retains overriding control, and that parental responsibility exists for the benefit of the child.^23^ Whilst parents have the right to consent to their child’s medical treatment, this right is not absolute. It will not apply if the decision is not in the BI of the child. In a different context, Holmes J stated that ‘parents may be free to become martyrs themselves, but it does not follow they are free, in identical circumstances, to make martyrs of their children’.^24^ In *Gillick*, Lord Scarman stated that ‘parental right must be exercised in accordance with the welfare principle and can be challenged, even overridden, if it be not’.^25^

The power of the court to declare what action is in a child’s BI also contrasts with the public law position requiring that a threshold be met before the court has jurisdiction to determine whether a child should be taken into care. An order committing the child to the care of the local authority under section 31 of the Children Act 1989 cannot be made unless the child ‘is suffering or is likely to suffer SH’ and the harm or likelihood of harm ‘is attributable to a lack of adequate parental care or control’. But where the question or dispute concerns the private law issue of what medical treatment option is in the BI of the child, no such threshold applies.

A challenge was recently mounted to the jurisdiction of the High Court to hear a case about the treatment of a critically ill child in circumstances where the option preferred by parents would allegedly not amount to SH but was considered by clinicians to be contrary to the child’s BI. In *Yates & Anor v Great Ormond Street Hospital for Children NHS FT*, the Court of Appeal firmly rejected the challenge and reiterated that the child’s BI is ‘the established yardstick’.^26^ Subject to the Supreme Court reaching a different view, the law as it stands does not countenance a harm threshold.

Proponents of reform differ in nuance and approach but broadly seek to give greater authority to parents and reduce the power of HCPs and the state’s reliance on the BI standard which, because it is centred on the child’s overall interests and is child- and fact-specific, does not always provide a predictable outcome.^27^ If a harm threshold is normatively justified, that change might be brought about by Parliament. To date, legislative proposals have come to nought,^28^ but the public interest in the matter is such that section 177 of the Health and Care Act 2022 required the Secretary of State to arrange for a review into the causes of disputes. The Nuffield Council on Bioethics was commissioned by the Department of Health and Social Care to report on the matter. In its 2023 report, the Council acknowledged the ‘considerable debate’ generated by calls to adopt an SH criterion but in its brief consideration of the arguments for a new SH criterion, found no compelling reasons for change.^29^ We support that conclusion and seek to contribute to the literature that demonstrates the normative, jurisprudential and clinical inappropriateness of an SH criterion.

## III. DISPUTING CRITICISMS OF THE BI THRESHOLD

The case for giving parents discretion as to which treatment their seriously ill child has, so long as their choice does not cause them SH and is ‘good enough,’ rests on both the use of SH *and* a presumption of parental autonomy. So, we start with an explanation of why that presumption is needed and proceed to a criticism of it. We then examine three principal reasons given for favouring SH over BI: indeterminacy, moral pluralism and consistency.

In the subsequent section, we set out a case in defence of the BI criterion.

### A. Parental autonomy

The preference for SH over BI is usually taken as meaning this: Parental choices of medical treatment should prevail so long as they do not risk or cause the child SH, rather than their being required to agree to whatever treatment promotes the child’s BI. However, this construal of the claim does not follow simply from a preference for SH over BI. It only does so if we start from a defeasible presumption in favour of the parental choice. So, on the preferred view, if parents and doctors disagree as to which of two possible treatments is best but agree that neither is significantly harmful, the parental choice wins out over the doctors’ preference. But it does so only when the SH criterion is combined with the presumption in favour of parental choice. That means that, using the same circumstances—agreement that neither treatment is significantly harmful but disagreement as to which is best—in conjunction with a presumption in favour of the doctors, what *they* and not the parents choose as the treatment the child should receive would be decisive.

That is why it makes sense to see the claim made by those in favour of the SH criterion in terms of the ‘zone of parental discretion’ explicated and defended by Lynn Gillam. This zone, in her words, defines:

the ethically protected space where parents may legitimately make decisions for their children, even if the decisions are sub-optimal for those children (i.e. not absolutely the best for them). …. In this space, “good enough” parental decisions should be tolerated, until the point where they would cause harm to the child.^30^

The view that parents have a warranted right to make choices for their own children, constrained by SH, is sometimes just assumed or is taken as a basic fact about how parenthood is viewed. For instance, Julian Savulescu and Dominic Wilkinson seem simply to presume that parents must have the greater right to choose, or this is how our society judges the matter.^31^ Lynn Gillam similarly thinks the claim that ‘Parents have an ethical right to make medical decisions for their children, based on their own conception of the good life’ is not controversial and is ‘widely recognised’.^32^ Cressida Auckland and Imogen Goold cite the significant international media attention as evidence of a ‘substantial disjunction between what the legal position is and what many people believe it ought to be’ and consider this one of the justifications for questioning the settled approach.^33^

However, given the important justificatory role it has, parental autonomy needs to be defended and not simply presumed. We should also never assume that what society views as right is right. Even those who think parental autonomy uncontroversial will be prepared to offer arguments in its defence. These can take one of two forms. Either it is seen as based in a self-evidently warranted power of personal choice, or it is seen as justified by an appeal to what we can say about the parents’ relation to their child.

A prominent example of the first is the view that parental autonomy is just a form or extension of personal autonomy. The obvious reply is that personal autonomy is a right of choice over one’s own life, whereas parental autonomy is a right of choice over another’s (the child’s) life. And the child is neither an extension of nor the property of the parent. Since John Stuart Mill is generally viewed as the most influential defender of an ideal of individual autonomy (he never uses the term, but what he understands by personal liberty captures what is at stake here), it is worth quoting his pertinent criticism of those who might invoke an ideal of parental autonomy:

It is in the case of children that misapplied notions of liberty are a real obstacle to the fulfilment by the State of its duties. One would almost think that a man’s children were supposed to be literally, and not metaphorically, a part of himself, so jealous is opinion of the smallest interference of law with his absolute and exclusive control over them; more jealous than of almost any interference with his own freedom of action: so much less do the generality of mankind value liberty than power.^34^

Johan Christiaan Bester is thus right to criticize those like Douglas Diekema who invoke Mill’s harm principle in defence of the use of SH in medical decision making.^35^ However, the problem he identifies as a ‘category mistake’, namely that the relations between parents, doctors, and the child are of a different order to that between the state and the individual, can be more simply stated: the freedom of an individual to make choices as to how to lead their life does not extend to nor encompass a freedom to make parental choices, that is choices over another human being’s life. The conventional legal position we seek to defend conforms to this view. As Rachel Taylor states, ‘the first important principle of parental responsibility is that the parental role is one of responsibility to children rather than proprietary rights over them’.^36^

The other way in which parental autonomy is defended appeals to the special nature of the relationship between parent and child. It most often takes the form of an assertion that parents know their children better than and care for them more than anyone else. Douglas Diekema, for instance, asserts that since parents care about their children, ‘they will usually be better situated than others to understand the unique needs of their children, desire what’s best for their children, and make decisions that are beneficial to their children.’^37^ Diekema’s article and its key claim is much cited by those defending SH.^38^

This claim is also expounded by Allen Buchanan and Dan Brock in their influential account of who chooses what is done to whom and why.^39^ They consider who should be proxy decision makers for those unable to choose for themselves. They are not addressing the cases of disagreement between parents and doctors. However, their account is of obvious relevance to such cases because they defend a version of parental autonomy over children and do so in a clear and carefully constructed manner that merits critical analysis. They think parents should be the surrogate or proxy decision-makers for their children but that their choices should be guided by the best interests principle. This is important, as we shall see.

They suggest two ‘obvious and compelling’ reasons for giving parents the powers of surrogate decision making in respect of an incompetent child: that parents are those ‘most knowledgeable’ about the child’s good and also those ‘most concerned’ about the child’s good. These reasons are epistemic and motivational. However, they are neither as obvious nor as compelling as Buchanan and Brock urge. In respect of the epistemic reason, there is an important distinction to be made between knowing one’s child and knowing what is best for one’s child. It is easy to conflate the two. Doubtless, parents who live with their children and who undertake their everyday care have an exceptionally close enduring relationship with them. They will certainly know better than others what their children like and do not like, their beliefs, moods, unique traits, character quirks, preferences, and fears. However, knowing all of that does not mean that they know what is best for those children. Moreover, it is all too easy for the motivational commitment to one’s child, and investment in their welfare to cloud judgments of what is best. MacDonald J acknowledged this in the case of *Haastrup* when he spoke of the impact on parental decision making of ‘abiding love and fierce devotion and the amplifying effect on those emotions of the flattering voice of hope.’^40^

Why does good knowledge of one’s child not mean knowledge of what is good for one’s child? Because there is a gap between the former and the latter. This is in part a justificatory gap between what is essentially factual knowledge—of what a child likes and dislikes, of what makes them happy or unhappy—and what is a normative judgement—of what *ought* to be done to promote their BI. It is also a simple gap in factual knowledge: A parent need not know what food regimen, educational activities, and of course medical procedures and care work best for any child. And the gap can also be the simple but significant one between knowing what is best and knowing how to bring that about.

The second reason for thinking that parents are best placed to choose for their children, the motivational one, does not hold for all parents nor for all circumstances. Most parents are moved to do what they think is best for their children and they will on occasion be moved to act in extraordinarily selfless fashion. Yet, we need to acknowledge an important distinction. Being moved to do what you believe to be best is not being moved to do what is, in fact, best. That is true, however, strongly the belief is held. Sadly, it must also be acknowledged that a small if, nevertheless, not insignificant number act to harm their children.

Some also act on unreasonable if sincerely held beliefs as to what is best and in doing so harm their children. A rare, dramatic, if admittedly controversial, example is that of parents conscientiously insisting upon an extreme and rigid vegan diet for their children.^41^

Buchanan and Brock’s ‘compelling and obvious’ reasons for parental autonomy and the power of proxy choice are thus neither compelling nor evident. They also recognize that such autonomy must be exercised in the interests of the child. Parents, they say, have

no independent interest or right to decide for their children and to enforce their choice when the choice may not best serve their children’s welfare. Instead, it makes the parents’ claim to decide wholly dependent on their tendency to decide more closely in accordance with their children’s welfare.^42^

The claim that most parents do tend to choose what is best for their children is of course plausible and attractive. But it is a considerably weaker claim than one that asserts that parents are the most knowledgeable and the most concerned choosers for their own children. This is the claim made by Diekema and others who employ a principle of parental autonomy to support the SH criterion.^43^

Moreover, the weaker claim gets further plausibility from being conflated with two quite different claims. The first is that even if parents are not the best people to rear their children, it is better if the law presumes that they are. We may thus design policies in respect of children *as if* their parents are the best placed to choose for them. This presumption is defeasible and must allow that parents are sometimes and, in some circumstances, not best placed to make choices for their children. The relevance to medical decision making is both obvious and compelling.

The second claim to distinguish is that parents are better parents if *they* act on the presumption that they are. Often, we are better at doing things if we think that we are. Parenting may be an instance of a self-confirming belief of this kind. Yet, even if this is generally the case, it need not always be so.

Buchanan and Brock assert that parents have ‘no independent interest or right to decide for their children’. They may, nevertheless, have an interest in discharging the role of making decisions for the child solely in the light of what is best for the child. Harry Brighouse and Adam Swift give admirably clear expression to this claim:

The rights that parents have over their children are indeed justified by appeal to children’s interests—parents should have just those rights that it is in children’s interests for them to have. But parenting relationships—and hence the family itself—are justified in part by the fact that adults have an interest in playing that fiduciary role.^44^

Buchanan and Brock’s claim that parents can only choose for their children if they choose what is best for them would leave it unclear why adults would want to be parents. Brighouse and Swift assert that parents do have an interest in choosing for their children. But it is one of doing what is best for the child. Such a view echoes the principle entrenched in law that the parental role is one of responsibility to children rather than one based in ownership of the child or one that is an extension of personal autonomy.

### B. Indeterminacy

An argument sometimes used by defenders of the SH criterion is that BI is vague, indeterminate, and imprecise. This criticism is a standard one that predates the current debate around disagreements.^45^ Of course, it only has bite in the present context if it can be shown that the BI criterion is afflicted by an indeterminacy (or degree of indeterminacy) that does not afflict SH.^46^ However, many will feel obliged to concede that, as the title of Charles Foster’s piece on the matter has it, harm is as indeterminate as ‘BI’.^47^ Foster quotes with approval Giles Birchley who claims that the notion of harm is no less intrinsically indeterminate than that of BI.^48^ Indeed Dominic Wilkinson, a prominent defender of SH over BI, concedes that:

although the harm threshold may be vague, there is no reason to think that it is any more vague that the best interests test …. If the harm threshold were only being used in place of the best interests test because of its putative ease of use, or because the best interests were ‘vague’ – these objections would be particularly important. However, … there are several arguments in favour of the harm threshold that do not depend on it being clear or determinate.^49^

In short, SH is not to be preferred over BH because the former is less vague or unclear than the latter. So, what remains of the argument? For Wilkinson it is that the former displays a ‘moral uncertainty’ the latter does not. However, if attention is paid to how the terms are defined, this claim is considerably weakened if not entirely undercut. For harm is standardly defined in terms of interests. Joel Feinberg, for instance, influentially defines a harm as a setback to interests,^50^ so the same core concept is employed in both criteria. SH is defined as a setback to harms beyond a certain threshold of seriousness; BI is defined as the optimal promotion of interests.

Moreover—and critically in this context—Feinberg defines a harm in normative terms. First, the setback is a wrongful invasion or set-back of another’s interest.^51^ Secondly, as others have made explicit, the interest is one that the other has a right to.^52^ So the person who thwarts an attempted crime does not harm the would-be criminal. If interest, as used in both definitions of SH and BI, is not morally determinate neither criterion escapes the charge of moral indeterminacy.

If it is said that the indeterminacy results from the qualifying adjectives used in each criterion, then BI fares better than SH. For whereas one cannot promote interests beyond an optimal point (what is best), it need not be clear where the threshold is fixed whereby a setback to interests beyond that point is a significant one.

Finally, it may be said—as Foster does—that BI is more complex and harder to use than SH.^53^ Wilkinson explicitly discounts the idea that such a qualification—‘putative ease of use’—might help the argument in favour.^54^ He is right to do so. The criteria under review are used in decision making about what care and treatment a child ought to receive. This should not be a simple matter. For what is at stake is the life and well being of a child. The decision is not only momentous but will also involve a range of considerations, which must be properly understood, evaluated, and appropriately weighed in any overall assessment. It will certainly go beyond a simple medical appraisal and the courts have indeed insisted on a holistic approach to the welfare of a child:

in considering the best interests of this particular patient at this particular time, decision-makers must look at [the patient’s] welfare in the widest sense, not just medical but social and psychological.^55^

### C. Reasonable pluralism

A further argument for the preferability of SH overs BI appeals to the significance of the disagreement between parents and HCPs. Here, the background thought is to be found in influential accounts of ‘reasonable disagreement’. A liberal state should recognize that its citizens reasonably disagree about values. Indeed, for John Rawls, the fact of reasonable disagreement is an inevitable consequence of the guarantee to citizens of the deliberative freedoms of speech, thought, religion, and association.^56^ The liberal state is founded on a principle of equal respect for all persons. Thus, the state should abstain from showing a preference in its laws and policies for any set of values or ‘conception of the good’. All of this is extensively discussed in contemporary political philosophy.

However, reasonable pluralism as defined does not provide support for BI over SH in the adjudication of conflicts between parents and HCPs. In the first place, neither criterion is any more or less laden with some conception of the good. As we have seen the notion of interests common to both criteria involves a notion of what is legitimate. Interest is an inescapably normative concept.

Secondly, recognition of reasonable disagreement does not of itself give us a reason to defer to the parents. Wilkinson and Savulescu state that when there is reasonable disagreement between parents and HCPs with respect to the treatment of a child we should defer to the values of the parents whose child it is and gloss this as justified not because the parents are right but ‘*just* because there is reasonable disagreement’.^57^ The ‘just’ is emphazised because no reason is given as to why the mere fact of disagreement gives us any reason to favour either view in the disagreement. It does not do so at the Rawlsian level of disagreements between citizens in a liberal society; it does not do so at the level of disagreements between parents and HCPs about the treatment of a child.

Auckland and Goold talk about respect for the plurality of values as grounding a preference for parental choices. But they explain this as follows; ‘The value of according parents decisional autonomy may also be justified because it shows respect for pluralism. It enables parents to pass on particular views, values and religious commitments to their children.’^58^ However, deciding what treatment a child should have cannot be construed as analogous to the transmission to a child of values by the manner in which it is reared. As noted earlier, parental autonomy should not be construed as an extension of personal autonomy. Moreover, Rawlsian liberals disagree as to whether parents are permitted to pass on values to their children.^59^

Thirdly, reasonable pluralism is pluralism of values. But the disagreement between parents and HCPs need not be exclusively normative. Some disagreements between clinicians and patients are not about values (say, about those informing a view on the quality of a child’s life) but rather factual matters such as what has been diagnosed and what will be achieved by various treatment options. If the disagreement is about the latter, it is unclear why medical expertise should not be decisive.

### D. Consistency of public and private law

Some proponents of SH claim that because child protection in a liberal state uses SH or risk of such harm as the trigger for intervention, consistency requires that the same criterion is used in the case of medical treatment.^60^ As we explore below, a demand for consistency has force if the two contexts are analogous, but here there are important and relevant differences between them. Consider the following: In child protection cases intervention is triggered. By this is meant a radical and often enduring change in the rights and responsibilities of the parents. There may be ongoing monitoring of family life and a subsequent loss of what might be termed familial privacy. Parents may lose for a specified period, sometimes permanently, their rights over their child. Compare these two kinds of medical treatment case. In the first, parents have failed adequately to care for their child by not seeking medical treatment for a serious condition; or by giving their child health care that would not be regarded as acceptable by qualified medical professionals. For example, when Baby Y’s mother fabricated reports of her baby suffering pain and vomiting, caused him physical pain and interfered with his medical treatment, resulting in numerous interventions and months in hospital, the judge was satisfied that ‘the mother’s behaviour caused or was likely to cause Y to suffer significant physical and emotional harm’.^61^ In the second, parents have brought their child to the attention of health professionals and sought advice and treatment from them. They refuse to consent to such treatment as is advised. In the first kind of case child protection measures would likely be triggered. Yet, this is not the situation with cases that conform to the second category, where parents seek to do the best for their child but nonetheless risk acting against the child’s interests. Invoking the language of child protection and state intervention into family life to describe this second category is not only unhelpful but deeply misleading.

Related to this misuse of descriptive language is the idea that the state is somehow (in demanding the best medical choices for a child) intervening in a private space. For example, Auckland and Goold speak about the need, in making medical decisions, to balance ‘protecting’ the child and ‘preventing illegitimate incursions into family and private life’^62^ or, again, characterize overruling parental wishes as an ‘intrusion into the private decisions of parents.’^63^ The use of the term ‘private’ here is misleading because of a crucial ambiguity. ‘Private’ can be used literally to describe a space as one removed from the public gaze; or it can be used to characterize actions which ought not to be regulated or monitored by the state. Now of course private actions in the first sense may be thought private in the second. Thomas Nagel elegantly characterizes those aspects of human life (such as defecation and copulation), which he says are rightly protected ‘from the crippling effects of the external gaze.’^64^

However, it is false that all private (in the first descriptive sense) actions are private in the second (normative) sense. Rape, for example, can be accomplished apart from the public gaze but is rightly subject to legal regulation. Most relevantly, there are good reasons why family life should be conducted in private, save for those matters and occasions where a family may choose or must be out in public—holidays, trips to the cinema, schooling, etc. However, what may be done within a family does not escape public regulation by virtue of being done away from public scrutiny. Child abuse and neglect, or domestic violence, are not exempt from such scrutiny (and punishment) simply because they were done in the private space of a family home. The fact that many decisions parents make about their children are private in the descriptive sense is not a normative justification preventing public scrutiny of medical treatment decisions.

## IV. DEFENDING THE BI CRITERION

In this section, we defend the BI criterion from arguments that the SH threshold is of more practical relevance to HCPs and the courts.^65^ We begin by arguing that clinicians do not in practice employ an SH threshold to determine which cases will go to court. In subsequent sections, we then counter the argument that it would make no discernible practical or legal difference to assert an SH criterion, but would enhance logic, comprehension and legal consistency.

### A. Accommodating parental preferences within the BI criterion

What treatments clinical staff will offer in the BI of the child is a matter determined with reference to law, and ethical^66^ professional,^67^ and clinical^68^ guidance. HCPs will review the reasonable range of treatment options, though this might be narrow or even single. Where there are reasonable treatment alternatives, parental preferences will be relevant to the choice between them as an exercise of shared decision making.^69^ HCPs can lawfully deliver life-saving treatment without consent to preserve the life of a child,^70^ in which case they will explain their reasoning to the child and parents, acknowledge any objections, and where disagreement persists, will approach the court as soon as the child is stable. In less urgent situations, consent is required for treatment to be lawful and whilst this may come from the court in exceptional circumstances, it must usually come from the parents. Family-centred care is routine in all aspects of daily paediatric healthcare. As we describe above, in cases of disagreement second opinions, advice from a clinical ethics committee and mediation can help the parties to reach consensus.

It is clear that HCPs make considerable efforts to accommodate parental preferences. Our focus in this section is to counter arguments^71^ that empirical observations of such efforts demonstrate that HCPs are not choosing the option that will yield optimal outcomes as required by the BI test, and that, in practice, HCPs will apply an SH threshold before seeking judicial intervention.

Birchley interviewed clinicians to determine what ‘BI’ implies. He found there to be little explicit mention of ‘harm’,^72^ and more extensive reference to what is ‘best for the child’.^73^ He eloquently demonstrates that this involves nuanced considerations of the wishes of the family whilst managing any negative impact on the child.^74^ Thresholds are frequently applied that are ‘perhaps “totemic of harm’’’ such as pain and suffering.^75^

Given that maximizing good is at least not harming, it is uncontroversial that HCPs will balance the harm and benefits of various treatment options. ‘Best’ is more than not harming whilst failing to do what is best for the patient is not inevitably harming them. The HCPs’ assessment of BI will therefore include an assessment of the harms and benefits of possible treatment options, including those suggested by parents. It is not a matter simply of attending to SH.

Diekema criticizes the BI criterion on the basis that ‘Best interests all too frequently may be reduced to objective medical interests alone’,^76^ but the courts in England and Wales are clear that decision-makers must be guided by an overall assessment of BI. HCPs too should make an overall BI assessment when determining the interests of the child.^77^ This includes consideration of the impact on the child of going against the parental view. The child’s emotional interests may be served by a suboptimal clinical option if doing so will maintain trust and prevent the breakdown of the relationship between parents and HCPs. Woodhouse puts it in this way:

A truly child‐centred perspective would also expose the fallacy that children can thrive while their care givers struggle, or that the care giver's needs can be severed from the child's, which has led to the attitude that violence, hostility, and neglect toward the care giver are somehow irrelevant in the best interest calculus.^78^

Other factors too might influence the decision to go to court. The cost of court proceedings, media, and other external pressures and criticism are relevant considerations provided the first consideration is the BI of the child. So too, the GMC advises that the HCP’s commitment to the health, wellbeing and autonomy of parents is compliant with ethical principles provided it is commensurate with the child’s BI.^79^ If recourse to the courts can be avoided, without causing HCPs to act in a way that conflicts with their professional conscience then this is positive for all parties. Equally, once a case proceeds to court, reasonable variations in parenting are recognized, subject to the foundational principle of child law that responsibility for the upbringing of the child is a collaborative venture that is not subject to threshold criteria.^80^ As such, empirical evidence that parental views are often accommodated within clinicians’ or judicial assessments of the child’s BI does not prove that a BI criterion is not at play or that an SH threshold is employed by HCPs and the courts.

### B. The negative impact of an SH criterion

None of this rejects the notion that many of the cases that are referred to court would in fact meet the SH criteria. Victoria Butler-Cole KC has said:

[S]witching to a threshold of SH would not make any material difference to the judgments made by the courts: doctors tend not to refer disputes to the court unless they have strongly held views that continuing treatment, or failing to provide it, would be completely contrary to their duties to the child.^81^

On the other hand, Birchley has said:

Although it is possible that adopting the harm threshold could ultimately have minimal effects on decision-making in practice, it is equally possible that a switch to the harm threshold signal would involve radical changes. These may involve unintended consequences to parental autonomy, since the harm threshold may invite a much more clinically dominated approach to welfare than the current system. It may also open decision-making up to great conflicts, since limits in resources play a much more overt part in limiting children’s treatment.^82^

In the *Gard* case, the Court of Appeal accepted policy arguments put forward by Katie Gollop KC as to why an SH threshold would not justify its object. First, it would result in a significant rise in cases coming before the court with parents attempting to demonstrate that suboptimal alternative therapies would not cause the child SH.^83^ Secondly, it would conflict with judicial endorsement^84^ of guidance from the Royal College of Paediatrics and Child Health (RCPCH), *Making Decisions to Limit Treatment in Life-limiting and Life-threatening Conditions in Children: A Framework for Practice*. The guidance considers three sub-categories where quality of life is limited and where life prolonging treatment may not alleviate the burdens of illness or treatment. Each has equal weight. They include ‘… C: Lack of ability to benefit; the severity of the child’s condition is such that it is difficult or impossible for them to derive benefit from continued life.’ Applying the SH threshold, group C cases would potentially be excluded.^85^

Katie Gollop KC and Sarah Pope have further argued that an SH criterion would impact adversely on families, forcing HCPs to characterize their choices as harmful if they are to reach the appropriate threshold for the court to adjudicate the child’s BI.^86^ For example, a parent whose religious views dictate that suffering can serve as a test from which follows spiritual reward, might favour a treatment path that would satisfy the definition of SH whilst remaining fully committed to their child’s BI. Far from reducing conflict, the application of the threshold could drive an adversarial approach^87^ and exacerbate conflicts about scares resources.^88^ We doubt that the focus on specific medical issues rather than the risk posed by parents^89^ would alleviate this issue given the focus of the SH threshold on the rights of parents to decide up to the threshold.

### C. The SH criterion would conflict with professional responsibilities

Jo Bridgeman argues that in child treatment cases:

balanced against the claims to autonomy, self-determination, choice, exercise of rights, and fulfilment of responsibilities of parents are professional duties to the child and public responsibilities for the protection of child welfare.^90^

Building on this argument, in this section we examine the professional duties of HCPs in light of tort and criminal law principles to demonstrate the inappropriateness of an SH criterion from a perspective of legal ‘fit’.^91^ In practice, imposition of an SH threshold might result in the courts adopting a broad interpretation of SH in order to limit the scope of parental discretion.^92^ In this section, however, we focus on the possibility that it would result in expanded parental authority.

The argument that principles from different areas or types of law should fit the legal landscape in a manner that ensures that one does not subvert the other is distinct from the idea we rejected earlier that a public law principle (the SH criterion) should also apply in an allegedly analogous private law context. We argued that it is not, in fact, analogous. In this section, we argue that if one area of law is developed in a manner that undermines other areas of law this would have problematic implications for those other areas.

We advance two related arguments. The first is that a consumeristic focus on parental choice would be inappropriate insofar as it could conflict with principles of tort law that protect the relevance of clinical expertise when establishing which treatment options to offer a patient. The second is that an SH criterion could increase pressure on HCPs to accept treatment requested by parents that cannot be considered ‘proper medical treatment’ and would therefore conflict with established criminal law principles. Neither argument covers the totality of scenarios in which the SH criterion would apply, but they do establish a tension between its imposition and established legal principles.

We begin with an argument that a legal endorsement of consumeristic parental choice is not relevant to parental preferences for options that are not in the child’s BI. There are two reasons why the contrary might be supposed. To begin with, advocates of the SH criterion often point to the practical application of value judgments in medical treatment decisions as a reason for allowing parents to make decisions up to the threshold of SH.^93^ Additionally, the Supreme Court in *Montgomery v Lanarkshire HB* (*Montgomery*) endorsed the idea of ‘consumers exercising choices’ in a separate legal context of clinical negligence.^94^ The Court found that adult patients should be empowered to choose between reasonable treatment alternatives through the provision of information on material risk. A failure to do so would risk the HCP breaching their duty of care to the patient. Lady Hale said:

A patient is entitled to take into account her own values, her own assessment of the comparative merits of [different options], whatever medical opinion may say, alongside the medical evaluation of the risks … The medical profession must respect her choice, unless she lacks the legal capacity to decide.^95^

Leaving aside for now arguments made above that separate personal autonomy and parental autonomy, we can confidently assert that the law distinguishes between patient choices between clinically viable treatment options and the role of patient choice in deciding which treatment alternatives are clinically viable. *Montgomery* protects the former and there is no reason why that protection should not also extend to parents choosing between clinically viable treatment options for their child. But there is no corresponding duty on clinicians to accept an option desired by either an adult patient or a parent where the option is not clinically indicated. This was made clear with respect to adult patients in *McCulloch v Forth Valley Health Board*,^96^ where the Supreme Court confirmed that the reasonableness of the HCP’s selection of treatment alternatives is assessed according to the *Bolam* test of the reasonable doctor; whereas assessment of disclosure of information on risks of the reasonable alternative treatment will be assessed according to the *Montgomery* standard of the reasonable or particular patient. The Supreme Court held that:

The identification of which treatments are reasonable alternatives (i.e. clinically appropriate) is … a matter falling within medical expertise and professional judgment, and hence governed by the professional practice test.^97^

Amongst the court’s justifications for this position was the need to avoid ‘an unfortunate conflict in the doctor’s role’ if doctors were required to offer an alternative treatment notwithstanding their reasonable medical opinion that it is not appropriate.^98^ Whilst the patient may request treatment alternatives, the reasonableness of those from which the patient gets to choose is an assessment that requires clinical expertise. Reasonable alternatives are those that the HCP can justifiably and reasonably claim to be suitable alternatives on the particular facts.

Whilst *McCulloch* did not focus on cases where an adult patient requests a particular treatment, the judgment did assert the importance of consistency with medical professional guidance.^99^ General Medical Council (GMC) guidance on consent is compliant with the judgment in its insistence that treatment that is not in the clinical interests of the patient should not be offered. This is so even if it is requested, though discussion with the patient might serve to assess the patient’s needs which could potentially inform the clinical decision about which treatment options are suitable.^100^

Thus, we can make the relatively uncontroversial point that the focus on patient autonomy in *Montgomery* is not relevant to decisions about which treatment alternatives are clinically viable. More importantly, we can claim that an SH harm criterion could conflict with legal principles set out by the Supreme Court insofar far as it could create a zone in which the parent has extended control of what options are reasonable.

For those advocating an SH criterion, if a parent were to suggest an option that is not clinically indicated but does not cause SH, and provided HCPs could not justifiably refuse it on grounds of resources, clinicians could find themselves pressured to provide it or to continue existing treatment, against their view of what is clinically indicated. The clinician could in theory refuse, for the law will not countenance an HCP being required to treat against their judgement.^101^ But even then, if the parent is empowered to choose to the threshold of SH, there would be pressure to facilitate treatment by others, especially if the parent has found an HCP somewhere who is willing to treat.

The latter scenario deserves more consideration. The age of the internet has led to, sometimes crowdfunded, global searches for HCPs who might provide treatment sought by the parent. It is conceivable that treating HCPs might tolerate or even aid parents in this search if they reasonably consider the treatment on offer elsewhere but unavailable domestically to be in the child’s BI. We are not concerned with that eventuality here. The converse case is where the offer of treatment from elsewhere is considered by the treating HCP to be contrary to the child’s BI. In the case of *Haastrup*, MacDonald J said:

It would be extremely unfortunate if the standard response to applications of this nature was to become one of scouring the world for medical experts who simply take the view that the medical, moral or ethical approach to these issues in their jurisdiction, or in their own practice is preferable to the medical, moral or ethical approach in this jurisdiction.^102^

Currently, as Lady Hale has stated: ‘parents are not entitled to insist upon treatment by anyone which is not in their child’s best interests.’^103^ Treatment that conflicts with the treating clinicians’ views of what is in the child’s BI but does not meet the SH threshold is problematic in terms of its fit with broader legal principles if it cannot be considered a ‘reasonable treatment option’. What falls within this category is not defined simply by the availability of HCPs willing to treat. In England and Wales, it is governed by professional obligations, and in particular, by evidence-based medicine.^104^ A second opinion and offer of treatment, that is supported by an evidence base would be unlikely to be considered contrary to the child’s BI^105^ or indeed to result in an application to court for a declaration of BI. But an offer to take over care that conflicts with the treating clinicians’ professional views of what constitutes a reasonable treatment option could engage professional obligations to the patient, which encompass omissions as well as acts.^106^ In such circumstances, the imposition of an SH threshold could lead to clinicians relinquishing their patient to a treatment regimen that according to standards of evidence-based medicine, is unreasonable because it is not clinically indicated. As such, an SH threshold has potential to result in a subset of cases where parental discretion trumps an evidence-based assessment of what treatment options are clinically indicated. This is problematic for cases that fall into this bracket but do not reach the SH threshold because there is no opportunity to seek independent adjudication of the child’s BI.

To be clear, we are not suggesting that HCPs who fail to prevent treatment that is not clinically indicated or indeed those who offer such treatment are necessarily negligent. Rather, our point is to demonstrate the lack of fit between an SH criterion that would limit the relevance of professional expertise, and tort law principles recently approved by the Supreme Court that endorse HCPs’ professional expertise in the identification of reasonable treatment options. The BI criterion is more consistent with tort law principle as is recognizes HCP expertise in determining which options are clinically indicated^107^ and leaves open the option of independent arbitration by the court if the implications of that judgment for the BI of the child is disputed.

Relatedly, the SH criterion would also fit poorly with criminal law principles. In what follows, we build on Bridgeman’s persuasive argument that professional conscience will rule some options out, even if they are preferred by the parents,^108^ and we advance an argument that an SH criterion could require or facilitate treatment that is not legally ‘proper medical treatment’.

All medical treatment interventions are *prima facie* harmful and criminal if they cause actual bodily harm^109^ or more serious injury. Exceptions to the rule must fit within established categories, one of which was referred to in *R v Brown* as ‘proper medical treatment’.^110^ Bodily invasions that do not constitute ‘proper medical treatment’ come within the purview of the criminal law. In those cases, consent (whether of a patient or proxy such as a parent) will not serve as a defence to the crime. Cases are few, but the principle is demonstrated in *R v BM*,^111^ where the removal of an ear, splitting of a tongue, and removal of a nipple from vulnerable customers by a body modification practitioner was unlawful, notwithstanding the customers’ consent. This was not ‘proper medical treatment’: the treatment was medically unnecessary, clinical standards of cleanliness were not applied such that no ‘reputable surgeon’ would have performed the procedures,^112^ and had they done so it would have constituted an assault.

There is not a body of case law defining ‘proper medical treatment’, particularly in the context of children, but we will argue that it fits treatment that can reasonably be considered to be in a child’s BI and does not fit treatment that does not cause the child SH. If we are successful, then a doctor could not lawfully treat in circumstances where they consider the treatment to be contrary to the child’s interests, just because doing so would not cause the child SH. There is a risk that such treatment would not constitute ‘proper medical treatment’.

The Law Commission considered the scope of ‘proper medical treatment’ in their consultation paper *Consent in the Criminal Law* in the context of recommendations about potential codification of the law.^113^ It set out nine pertinent issues to the medical exemption including ‘that the treatment is for a therapeutic purpose …’, ‘that if the person … is under 18, a lawful consent has otherwise been given’, ‘that the treatment is for the patient’s benefit’, and ‘that the treatment … involves a risk of harm that is not disproportionate to the expected benefits’.^114^ The latter criterion requires that the risk of harm is commensurate to the benefits rather than imposing a threshold of harm, and the penultimate criterion asserts that proper medical treatment is treatment that benefits the patient. The Law Commission points out that in *Bravery v Bravery*, Denning LJ found no exception in a situation where surgery was conducted ‘with consent but without just cause and excuse’,^115^ and recognized that what constitutes just cause and excuse is likely to change over time as attitudes evolve.^116^ It also recognized that the criminal law focus is relevant not just to surgery but also other aspects of ‘medical treatment and care’ including procedures for purposes of treatment, care, diagnosis, or prevention of disease.

Though the law was not subsequently codified, the Law Commission report has been influential in case law.^117^ The ambits of ‘proper medical treatment’ remain poorly defined but the GMC interprets the law and guidance to require that treatment of adults is conducted in their clinical interests.^118^ In the case of children, the GMC, in common with the law, endorses a more global assessment of the child’s BI. Consequently, if an HCP, having taken into account their professional and ethical duties, decides reasonably that a treatment suggested by parents is not in the child’s BI, then we would submit that the HCP cannot assume the treatment is ‘proper medical treatment’.^119^

If the law were changed to impose an SH criterion would the scope of ‘proper medical treatment’ simply change with it? We think this would be problematic. The threshold for treatment or other *prima facie* harmful physical interference to constitute a crime is actual bodily harm. Whilst we have argued that the concept of harm is no more determinate than BI, it seems reasonable to assume that SH requires more than mere ‘actual’ bodily harm, both because the SH is global rather than focussed on the physical aspects of the intervention and because ‘significant’ denotes more than the mere occurrence of harm. Diekema, for example, sets the threshold at damage to basic needs.^120^ He builds on Lainie Ross’s model of constrained parental autonomy that allows parents to choose how best to promote the overall welfare of their family, provided the basic needs of each child are protected allowing the children to become autonomous adults.^121^ Tolerable harm would include harm that increases the likelihood of serious harm compared to other options. We can conclude that treatment might not be ‘proper medical treatment’ if it causes actual bodily harm, even if the harm does not constitute ‘significant harm’.

As we have seen, whilst the court can rule out certain treatments as contrary to a child’s BI, it will not require a doctor to treat. Where a court determines that a certain treatment is in a patient’s BI, however, it can create a collective duty to treat and alleviate the moral distress of doing so in light of the independent view that the treatment can be conceived as proper.^122^ A judicial assertion that a treatment would not cause SH would not be effective in this regard. It would not demonstrate that the treatment is proper because it might still cause a degree of harm that requires justification.

In conclusion, insofar as an intervention could cause actual bodily harm, consent is not a valid defence unless the treatment constitutes ‘proper medical treatment’, which is unlikely if the treatment is not in the BI of the child. We are not suggesting that any treatment that is not reasonably considered to be in the child’s BI would result in criminal prosecution. The public interest in such a prosecution would be dubious. Additionally, the relevance of the crime is restricted to interventions that cause actual bodily harm, so does not include refusal of treatment, withholding treatment or any intervention that falls short of actual bodily harm. What our analysis does suggest is that a threshold allowing parents to control treatment options to the extent that they do not constitute SH would conflict with both tort and criminal law principles.

## V. CONCLUSION

We have countered four arguments in favour of the imposition of an SH threshold. We deny that it is the logical response to either reasonable disagreement of values or to the harm threshold that applies in care proceedings. We dispute the notion that it would enhance determinacy more effectively than the BI test and challenge the relevance of parental autonomy when parents choose in a way that is contrary to the child’s BI.

In defence of the status quo, we have argued that it is commensurate with the BI test that HCPs and courts assessing a child’s overall BI are strongly influenced by the views of parents where they comply with the child’s welfare and with professional conscience. It is equally important that where matters are finely balanced or the way forward preferred by parents would conflict with the professional duties of clinicians, that recourse be had to dispute resolution and ultimately the courts. In *Burke*, Lord Phillips MR was clear that ‘Once a patient is accepted into a hospital, the medical staff come under a positive duty at common law to care for the patient.’^123^

The sort of case where clinical evidence that the preferred parental approach would not cause SH might involve novel treatments, or continuation of treatment in cases where it is considered futile and there is no evidence that the child will suffer pain, for example. SH criteria are likely to have one of two implications. Victoria Butler-Cole KC envisages that it would result in an expansive judicial interpretation of harm:

A generous approach to the concept of ‘harm’ would inevitably be taken in order that such disputes could be resolved -even in cases where a child’s ability to experience pain and suffering, and thus to be harmed in the usual sense of the word, was severely compromised or absent.^124^

The practical implications of this could, as we examine above, aggravate the potential for breakdown of relations between clinicians and parents.

Alternatively, it could result in greater deference to parental autonomy. Up to the threshold, HCPs’ ability to seek guidance from the court would be driven by the views of parents rather than the needs of the child. There is potential for conflict with professional obligations, for ‘When treating children and young people, doctors must also consider parents and others close to them; but their patient must be the doctor’s first concern.’^125^ It would result in a poor fit with both criminal law principles governing an exception to battery in the case of ‘proper medical treatment’ and tort law principles emphasizing professional autonomy in the identification of clinically appropriate treatment options.

## Footnotes

1 See eg, DS Diekema, ‘Parental Refusal of Medical Treatment: The Harm Principle as Threshold for State Intervention’ (2004) 25 Theoretical Medicine and Bioethics 243; S Elliston, *The Best Interests of the Child in Healthcare* (Routledge Cavendish 2007); L Gillam, ‘The Zone of Parental Discretion: An Ethical Tool For Dealing with Disagreement between Parents and Doctors about Medical Treatment for a Child’ (2016) 11 Clinical Ethics 1; R Gillon, ‘Why Charlie Gard’s Parents Should have been the Decision Makers about their Son’s Best Interests’ (2018) 44 Journal of Medical Ethics 461; C Auckland and I Goold, ‘Parental Rights, Best Interests and Significant Harms’ (2019) 11 Cambridge Law Journal 287; D Wilkinson, ‘In Defence of a Conditional Harm Threshold Test for Paediatric Decision-Making’ in I Goold, J Herring and C Auckland (eds), *Parental Rights, Best Interests and Significant Harms* (Hart 2019), ch 5; D Wilkinson and J Savulescu, *Ethics, Conflict and Medical Treatment for Children: From Disagreement to Dissensus* (Elsevier 2019).

2 RJ McDougall and L Notini, ‘Overriding Parents’ Medical Decisions for their Children: A Systematic Review of Normative Literature’ (2014) 40 Journal of Medical Ethics 448.

3 Auckland and Goold (n 1) 290.

4 Diekema (n 1); Gillam (n 1) 2.

5 Auckland and Goold (n 1) 288, 300; Wilkinson and Savulescu (n 1) 14.

6 Wilkinson and Savulescu (n 1) 93.

7 Diekema (n 1); Wilkinson and Savulescu (n 1) 93.

8 J Bridgeman, *Medical Treatment of Children and the Law—Beyond Parental Responsibilities* (Routledge 2021), 23.

9 Our focus is on children who are not able to consent for themselves.

10 But see V Neefjes, ‘Can Mediation Avoid Litigation in Conflicts about Medical Treatment for Children? An Analysis of Previous Litigation in England and Wales’ (2023) 108 Archives of Disease in Childhood 715.

11 J Brierley, E Cave and D Archard, ‘Ethical Advice in Paediatric Care’ (2022) 107 Archives of Disease in Childhood e18.

12 V Larcher and J Brierley, ‘Second Medical Opinions in Paediatric Practice; Proposals for a Framework for Best Practice’ (2020) 105 Archives of Disease in Childhood 213.

13 See Nuffield Council on Bioethics, *Disagreements in the Care of Critically Ill Children* (NCOB 2023); L Austin and R Huxtable, ‘Resolving Disagreements about the Care of Critically Ill Children’ in I Goold, J Herring and C Auckland (eds), *Parental Rights, Best Interests and Significant Harms* (Hart 2019) ch 11.

14 For discussion of the legal mechanisms see R George, ‘The Legal Basis of the Court’s Jurisdiction to Authorise Medical Treatment of Children’ in I Goold, J Herring and C Auckland (eds), *Parental Rights, Best Interests and Significant Harms* (Hart 2019) 67–85. For an endorsement of the court’s role see S Fovargue, ‘Preserving the Therapeutic Alliance: Court Intervention and Experimental Treatment Requests’ in I Goold, J Herring and C Auckland (eds), *Parental Rights, Best Interests and Significant Harms* (Hart 2019) ch 8.

15 It is within the power of the court to embargo a particular form of treatment: *Yates & Anor v Great Ormond Street Hospital for Children NHS FT & Anor* [2017] EWCA Civ 410, [88] (McFarlane LJ) but the court will not require a doctor to treat against their judgment: *Re J (A Minor) (Wardship: Medical Treatment*) [1993] Fam 15, 26H; approved in *Aintree University Hospital NHS FT v James* [2013] UKSC 67, [18].

16
*Nottingham University Hospitals NHS Foundation Trust v Gregory & Ors* [2023] EWHC 2556 (Fam); [2023] EWCA Civ 1262.

17
*Yates* (n 15).

18
*King’s College Hospital NHS Foundation Trust v Haastrup* [2018] EWHC 127 (Fam).

19
*Barts NHS FT v Raqeeb* [2019] EWHC 2530 (Fam).

20
*Manchester University NHS FT v Fixsler and Ors* [2021] EWHC 1426 (Fam).

21 The Criminal and Civil Legal Aid (Amendment) Regulations 2023, SI 2023/756 amending the Civil Legal Aid (Financial Resources and Payment for Services) Regulations 2013, SI 2013/480.

22 s 3(1): ‘In this Act “parental responsibility” means all the rights, duties, powers, responsibilities and authority which by law a parent of a child has in relation to the child and his property.’

23
*Gillick v West Norfolk AHA* [1986] AC 112, 170 (Lord Fraser); *Evans v Alder Hey Children’s NHS Foundation Trust* [2018] EWCA Civ 805, [51]–[52].

24
*Prince v Massachusetts* 321 US 158, 165 (1944), cited in *Re E (A Minor) (Wardship: Medical Treatment)* [1993] 1 FLR 386, 391 (Ward J).

25
*Gillick v West Norfolk AHA* [1986] AC 112, 185 (Lord Scarman).

26
*Yates* (n 15) [74] (McFarlane LJ).

27 See eg, J Brierley, J Linthicum and A Petros, ‘Should Religious Beliefs be Allowed to Stonewall a Secular Approach to Withdrawing and Withholding Treatment in Children?’ (2013) 39 Journal of Medical Ethics 574, 576.

28 Efforts to introduce Charlie’s Law (<https://thecharliegardfoundation.org/about/what-is-charlies-law/> accessed 9 November 2023) include a Private Member’s Bill introduced by Bambos Charalambous called the Children (Access to Treatment) Bill 2021, and amendment 287 to the Health and Care Bill 2021 proposed by Baroness Finlay of Llandaff entitled ‘dispute resolution in children’s palliative care’: ‘4(b) the court may not make any order that would prevent or obstruct the parent from pursuing proposals for obtaining disease-modifying treatment for the child (whether in the UK or elsewhere) unless the court is satisfied that the proposals—(i) involve a medical institution that is not generally regarded within the medical community as a responsible and reliable institution, or (ii) pose a disproportionate risk of significant harm to the child.’ The amendment was rejected subject to s 177 referred to below.

29 Nuffield Council on Bioethics (n 13).

30 Gillam (n 1) 2.

31 Wilkinson and Savulescu (n 1) 59.

32 Gillam (n 1) 7.

33 Auckland and Goold (n 1) 290.

34 JS Mill, ‘On Liberty’, in S Collini (ed), *On Liberty and Other Writings* (Cambridge [1859]1989) 105, ch 5.

35 JC Bester ‘The Harm Principle Cannot Replace the Best Interest Standard: Problems with Using the Harm Principle for Medical Decision Making for Children’ (2018) 18 American Journal of Bioethics 9, 13.

36 R Taylor, ‘Parental Decisions and Court Jurisdiction: Best Interests or Significant Harm?’ in I Goold, J Herring and C Auckland (eds), *Parental Rights, Best Interests and Significant Harms* (Hart 2019) ch 3.

37 Diekema (n 1) 244.

38 For instance, Auckland and Goold (n 1) 299.

39 A Buchanan and D Brock, *Deciding for Others: The Ethics of Surrogate Decision Making* (Cambridge 1989) 136.

40
*Raqeeb* (n 19), [1].

41 See eg, G Massie, ‘Vegan Mother Jailed for Life after 18-Month-Old Son Starved to Death on Diet of Raw Fruits and Vegetables’ *The Independent* (London 31 August 2022); H Brewis, ‘Swedish Parents Jailed for Putting their Baby on Vegan Diet that Nearly Killed Her’ *The Standard* (London 24 May 2019).

42 Buchanan and Brock (n 39) 233.

43 Diekema (n 1) 244; Auckland and Goold (n 1) 298–99.

44 H Brighouse and A Swift, *Family Values: The Ethics of Parent-Child Relationships* (Princeton 2014) 54.

45 EK Salter, ‘Deciding for a Child: A Comprehensive Analysis of the Best Interest Standard’ (2014) 33 Theoretical Medicine and Bioethics 179.

46 SK Shah, AR Rosenberg and DS Diekema, ‘Charlie Gard and the Limits of the Harm Principle—Reply’ (2018) 172 Journal of the American Medical Association Paediatrics 301.

47 C Foster, ‘Harm: As Indeterminate as “Best Interests” but Useful for Triage’ (2016) 42 Journal of Medical Ethics 121.

48 G Birchley, ‘Harm is all you Need? Best interests and Disputes about Parental Decision-Making’ (2016) 42 Journal of Medical Ethics 111.

49 Wilkinson (n 1) 10–11.

50 J Feinberg, *The Moral Limits of the Criminal Law*, *Volume 1*: *Harm to Others* (New York 2014).

51 ibid 105–06.

52 A von Hirsch, ‘Injury and Exasperation’ (Review of Volumes One and Two of Feinberg, *The Moral Limits of the Criminal Law*) (1986) 84 Michigan Law Review 701.

53 Foster (n 47) 121, 122.

54 Wilkinson (n 1) 11.

55
*Aintree University Hospitals NHS FT v James* [2013] UKSC 67, [39] (Lady Hale) (a case on adults lacking mental capacity that is relevant to the global assessment of children’s best interests: see for example *E & F (Minors: Blood Transfusion)* [2021] EWCA Civ 1888 [49] (McFarlane, P)).

56 J Rawls, *Political Liberalism* (Columbia University Press 1993), Lecture II ‘The Powers of Citizens and Their Representation’.

57 Wilkinson and Savulescu (n 1) 164; emphasis added.

58 Auckland and Goold (n 1) 300–01.

59 M Clayton, *Justice and Legitimacy in Upbringing* (Oxford 2006).

60 As raised by counsel in *Yates* (n 15) [65]. And see discussion in Auckland and Goold (n 1) 314 and Wilkinson (n 1) 92.

61
*Y (A Child) Fact Finding: Fabricated Illness)* [2018] EWHC 4020 (Fam), [296] (Knowles J). Consider also the case of Ashya King that involved both private and public matters, the latter being triggered when parents seek to remove a child from hospital to access alternative care. See *Re King* [2014] EWHC 2964 (Fam).

62 Auckland and Goold (n 1) 289.

63 ibid 293.

64 T Nagel, ‘Concealment and Exposure’ in *Concealment and Exposure, and Other Essays* (OUP 2002) 15.

65 Diekema (n 1) 248.

66 See eg, Nuffield Council on Bioethics, *Critical Care Decisions in Fetal and Neonatal Medicine: Ethical Issues* (Nuffield Council on Bioethics 2006); Nuffield Council on Bioethics, *Children and Clinical Research: Ethical Issues* (Nuffield Council on Bioethics 2014).

67 Such as GMC, *0-18 Years: Guidance for All Doctors* (GMC 2007, updated 2018); V Larcher and others, ‘Making Decisions to Limit Treatment in Life-Limiting and Life-Threatening Conditions in Children: A Framework for Practice’ (2015) 100 Archives of Disease in Childhood s1.

68 For eg, RCPCH, ‘Clinical Guidelines’ <https://www.rcpch.ac.uk/resources/clinical-guidelines> accessed 9 November 2023.

69 NHS England, ‘Shared Decision-Making’ (2019) <https://www.england.nhs.uk/personalisedcare/shared-decision-making/> accessed 9 November 2023.

70
*Re F* [1989] 2 All ER 545. And see P Aubugeau-Williams and J Brierley, ‘Consent in Children's Intensive Care: The Voices of the Parents of Critically Ill Children and Those Caring for Them’ (2020) 46(7) Journal of Medical Ethics 482.

71 See Diekema (n 1); Wilkinson and Savulescu (n 1) 93.

72 Birchley (n 1), ‘The Harm Threshold: A View from the Clinic’ in I Goold, J Herring and C Auckland (eds), *Parental Rights, Best Interests and Significant Harms* (Hart 2019) 115: ‘“Harm” was mentioned only seven times in over 50 hours of interviews’.

73 ibid 120.

74 ibid 122.

75 ibid 122.

76 Diekema (n 1) 247.

77 See criticism on this point of the *Raqeeb* judgment (n 19) in E Cave, J Brierley and D Archard, ‘Making Decisions for Children—Accommodating Parental Choice in Best Interests Determinations’ (2020) 28 Medical Law Review 183. This was resolved in line with their criticism in *Birmingham Women's and Children's NHS Foundation Trust v JB* [2020] EWHC 2595 (Fam), [30] (Hayden J); *Fixsler* (n 20) [61] (MacDonald J).

78 BB Woodhouse, ‘Hatching the Egg: A Child‐Centered Perspective on Parents' Rights’ (1993) 114 Cardozo Law Review 1747, 1825.

79 See eg, GMC (n 67) 4: ‘When treating children and young people, doctors must also consider parents and others close to them; but their patient must be the doctor’s first concern.’

80 See Taylor (n 36) 65.

81 In ‘Foreword’ to I Goold, J Herring and C Auckland (eds), *Parental Rights, Best Interests and Significant Harms* (Hart 2019) vi.

82 Birchley (n 72) 132.

83
*Yates* (n 15) [72] (McFarlane LJ).

84
*Re A (HR)* [2016] EWCA Civ 759.

85
*Yates* (n 15) [77] (McFarlane LJ).

86 K Gollop and S Pope, ‘Charlie Gard, Alfie Evans and R (A Child): Why a Medical Treatment Significant Harm Test Would Hinder not Help’ (22 May 2018) <http://www.transparencyproject.org.uk/charlie-gard-alfie-evans-and-r-a-child-why-amedical-treatment-significant-harm-test-would-hinder-not-help/accessed> 9 November 2023.

87 A point amplified by Taylor (n 36) 61: ‘it would be unnecessarily cruel and combative to require loving and sincere parents to defend themselves against a test based on harm’.

88 Birchley (n 72) 129–30.

89 See Wilkinson (n 1) 96.

90 Bridgeman (n 8) 23.

91 R Dworkin, ‘Hard Cases’ (1975) 88 Harvard Law Review 1057, reprinted in R Dworkin, *Taking Rights Seriously* (HUP 1977) ch 4.

92 See V Butler Cole in ‘Foreword’ to I Goold, J Herring and C Auckland (eds), *Parental Rights, Best Interests and Significant Harms* (Hart 2019).

93 For eg, Auckland and Goold (n 1) 288.

94
*Montgomery v Lanarkshire* [2015] UKSC 11, [75].

95 ibid [115].

96 [2023] UKSC 26.

97 ibid [64].

98 ibid [71].

99 ibid [67]–[70].

100 GMC, *Decision Making and Consent* (GMC 2020) para 49. And see AI Sarela, ‘Re-thinking Consent for Treatment: Clinical Interests and the Public Interest’ (*Blog of the Journal of Medical Ethics*, 7 March 2023) <https://blogs.bmj.com/medical-ethics/2023/03/07/re-thinking-consent-for-treatment-clinical-interests-and-the-public-interest/>

101
*Re J* (n 15) 26H.

102
*King’s College Hospital NHS Foundation Trust v Haastrup* [2018] EWHC 127 (Fam), [83].

103 In the matter of Charlie Gard: Lady Hale’s Explanation of the Supreme Court’s Decision, as delivered in court on 8 June 2017 <https://www.supremecourt.uk/news/permission-to-appeal-hearing-in-the-matter-of-charlie-gard.html> accessed 9 November 2023.

104 GMC, *Good Medical Practice* (GMC 2024) para 7(e). And see AI Sarela, ‘The Test of Availability of Medical Treatment’ (2023) 23 Medical Law International 109.

105
*Raqeeb* (n 19) [178] (MacDonald J).

106
*R (Burke) v GMC* [2005] EWCA 1003, [32].

107 As mentioned above, there is scope for consideration of the patient’s/parent’s views in this analysis.

108 J Bridgeman, ‘Beyond Best Interests: A Question of Professional Conscience?’ in I Goold, J Herring and C Auckland (eds), *Parental Rights, Best Interests and Significant Harms* (Hart 2019) ch 7; And see Bridgeman (n 8).

109 Under the Offences Against the Person Act 1861, s 47: ‘Whosoever shall be convicted upon an indictment of any assault occasioning actual bodily harm shall be liable to imprisonment for a term not exceeding 7 years; and whosoever shall be convicted upon an indictment for a common assault shall be liable, at the discretion of the court, to be imprisoned for any term not exceeding two years.’

110
*R v Brown & Ors* [1993] UKHL 19, [1994] 1 AC 212, 266F-G. And see confirmation in *Airedale NHS Trust v Bland* [1993] AC 789, p 891 F.

111
*R v BM* [2018] EWCA Crim 560.

112 [2018] EWCA Crim 560, [19] (Lord Burnett LCJ)

113 Law Commission Consultation Paper No 139, Part VIII <https://www.lawcom.gov.uk/app/uploads/2016/08/No.139-Criminal-Law-Consent-in-the-Criminal-Law-A-Consultation-Paper.pdf> accessed 9 November 2023.

114 Law Commission Consultation Paper No 139, 8.12.

115 [1954] 1 WLR 1169; ibid 8.23.

116 Law Commission (n 114) 8.26.

117 See eg, *R v BM* [2018] EWCA Crim 560 [24] (Lord Burnett).

118 GMC (n 100).

119 Note that the law makes similar requirements of HCPs in the context of adults who lack capacity: Mental Capacity Act 2005, ss 4 and 5. s 5(1)(b)(ii) requires that the decision maker reasonably believes that the act that the patient lacks capacity to consent to is in the patient’s best interests.

120 Diekema (n 1) 251.

121 Lainie F Ross, *Children, Families, and Health Care Decision-Making* (OUP 1998) 131–41.

122 See E Cave, ‘Selecting Treatment Options and Choosing Between Them: Delineating Patient and Professional Autonomy in Shared Decision-Making’ (2020) 4 Health Care Analysis 4, 15.

123
*R (Burke) v GMC* [2005] EWCA 1003, [32].

124 In ‘Foreword’ to I Goold, J Herring and C Auckland (eds), *Parental Rights, Best Interests and Significant Harms* (Hart 2019) vi.

125 GMC (n 67) para 4.

